# The role of the microbiome for human health: from basic science to clinical applications

**DOI:** 10.1007/s00394-018-1703-4

**Published:** 2018-05-10

**Authors:** M. Hasan Mohajeri, Robert J. M. Brummer, Robert A. Rastall, Rinse K. Weersma, Hermie J. M. Harmsen, Marijke Faas, Manfred Eggersdorfer

**Affiliations:** 1 DSM Nutritional Products Ltd, Kaiseraugst, Switzerland; 2 University of Zurich, Irchel, Zurich, Switzerland; 3 Örebro University, School of Medical Sciences, Örebro, Sweden; 4 Department of Food and Nutritional Sciences, University of Reading, Reading, UK; 5 Department of Gastroenterology and Hepatology, University of Groningen and University Medical Center Groningen, Groningen, The Netherlands; 6 Department of Medical Microbiology, University Medical Center Groningen, Groningen, The Netherlands; 7 Department of Pathology and Medical Biology, University Medical Center Groningen, Groningen, The Netherlands

**Keywords:** Microbiota, Gut, Prebiotics, Probiotics, Vitamins, Colonic fermentation, Inflammatory bowel disease, Irritable bowel syndrome, Gut-brain axis, Obesity

## Abstract

The 2017 annual symposium organized by the University Medical Center Groningen in The Netherlands focused on the role of the gut microbiome in human health and disease. Experts from academia and industry examined interactions of prebiotics, probiotics, or vitamins with the gut microbiome in health and disease, the development of the microbiome in early-life and the role of the microbiome on the gut–brain axis. The gut microbiota changes dramatically during pregnancy and intrinsic factors (such as stress), in addition to extrinsic factors (such as diet, and drugs) influence the composition and activity of the gut microbiome throughout life. Microbial metabolites, e.g. short-chain fatty acids affect gut–brain signaling and the immune response. The gut microbiota has a regulatory role on anxiety, mood, cognition and pain which is exerted via the gut–brain axis. Ingestion of prebiotics or probiotics has been used to treat a range of conditions including constipation, allergic reactions and infections in infancy, and IBS. Fecal microbiota transplantation (FMT) highly effective for treating recurrent *Clostridium difficile* infections. The gut microbiome affects virtually all aspects of human health, but the degree of scientific evidence, the models and technologies and the understanding of mechanisms of action vary considerably from one benefit area to the other. For a clinical practice to be broadly accepted, the mode of action, the therapeutic window, and potential side effects need to thoroughly be investigated. This calls for further coordinated state-of-the art research to better understand and document the human gut microbiome’s effects on human health.

## Introduction

The University Medical Center Groningen (UMCG) in The Netherlands organizes annual symposia within the compass of medicine and nutrition, as part of its Healthy Ageing program. Previously published proceedings of these symposia have examined the relationship of nutrients with lifelong health and disease [1], with healthy aging [2], with malnutrition and obesity [3], and with nutrient–drug interactions [4].

The 2017 annual meeting at the UMCG focused on the role of the gut microbiome in human health and disease. The symposium, which brought together experts from academia and industry, examined interactions of prebiotics, probiotics or vitamins with the gut microbiome. The panel discussed the role of the microbiome on various aspects of healthy and diseased subjects throughout lifespan. In the context of disease, the symposia focused on two main intestinal conditions: inflammatory bowel disease (IBD), manifesting as Crohn’s disease (CD) or ulcerative colitis (UC); and irritable bowel syndrome (IBS). Moreover, the various benefits of prebiotics on human health, the microbiome–nutrient interaction and the role of vitamins in promoting the selective growth of microbes in the gut as well as determinants of the development of a healthy microbiome were presented and discussed intensively. Last but not least, the panel discussed how the brain and the microbiome may affect and control each other’s functions and the implications of such communication for treating or preventing the brain-related functional decline during aging.

It is worth noting that the terms microbiota and microbiome are frequently used interchangeably and this also applies here. Strictly speaking, however, microbiota is defined as the microbial taxa associated with complex organisms such as humans, whereas microbiome is the catalogue of these microbes and their genes [5]. The totality of data suggests great promise for use of pre- and probiotics in promoting general health and treating human diseases.

## Prebiotic interactions with the microbiome

Dietary prebiotics have been defined as “a selectively fermented ingredient that results in specific changes in the composition and/or activity of the gastrointestinal microbiota, thus conferring benefit(s) upon host health” [6]. This definition has been subjected to debate as it focuses largely around the need for selective metabolism. An alternative definition which includes the mechanism of action has been established recently in a consensus statement [7]. The expert panel revised the definition of a prebiotic as “a substrate that is selectively utilized by host microorganisms conferring a health benefit”. This updated definition still requires a selective microbiota-mediated mechanism to be defined as a prebiotic.

**Fig. 1:**
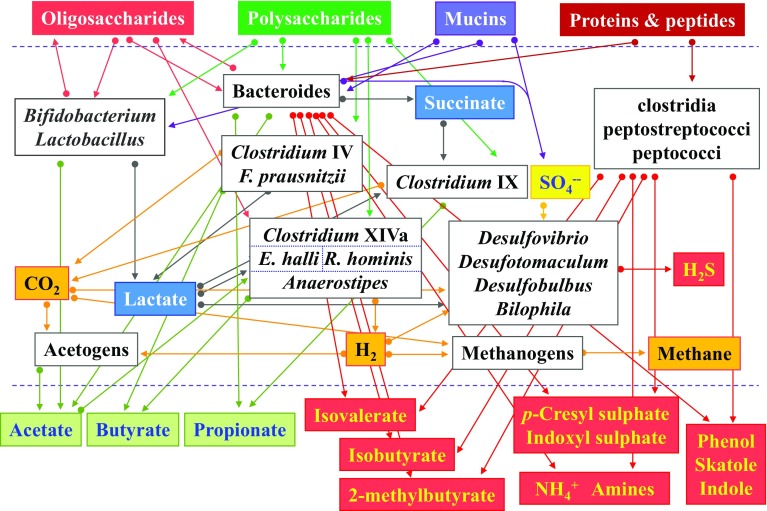
Fermentation and gut microbiota. The figure shows the principle sources of nutrition entering the human colon at the top and the principle metabolic outputs at the bottom. Arrows indicate known cross-feeding relationships between the principle microbial groups present. Metabolites in green boxes are believed to be health-positive while those in red boxes are potentially harmful. Gaseous products are in orange boxes and the most significant intermediate products of metabolism are in blue

Fermentation of dietary prebiotics in the gut involves metabolic cross-feeding where the products of fermentation by one or more bacterial species provide the substrate(s) for other bacterial species (Fig. 1) [8]. This complex cooperative activity of the gut microbiota is essential for good health [8, 9]. Bacterial fermentation of amino acids and proteins, which occurs mainly in the distal colon, generates a range of metabolites, many of which have a toxic potential. These include hydrogen sulphide, branched-chain fatty acids (BCFAs), phenol, indole, p-cresol, indoxylsulfate, p-cresylsulfate, and ammonia [10–12]. Even if also present in the healthy colon, it must be noted, however, that we currently have a very poor understanding of the concentrations of microbial metabolites in the human colon [12].

Several studies have demonstrated modulation of colonic microbiota by prebiotic inulin or inulin-type fructans. Real-time polymerase chain reaction (PCR) identification of selected bacterial species in the feces of human volunteers after inulin ingestion showed that the prevalence of *Faecalibacterium prausnitzii* and two *Bifidobacterium* species, *B. adolescentis* and *B. bifidum*, increased significantly [13]. In a placebo-controlled study, dietary inulin-type fructans increased the relative abundance of *Bifidobacterium* spp. and *F. prausnitzii* in obese women [14]. In healthy adults with mild constipation, inulin-type fructans increased the relative abundance of *Anaerostipes, Bilophila* and *Bifidobacterium* in feces, and reduced the abundance of *Bilophila* [15]. Differences in selectivity for the fermentation of several carbohydrate substrates (lactulose, galacto-oligosaccharides, sugar beet pectin and apple fiber) were found between the microbiotas from lean and obese healthy subjects using an in vitro model (TIM-2) of the proximal colon, providing the evidence that the composition of the microbiota changes depending on the body mass index (BMI) in humans [16].

**Fig. 2:**
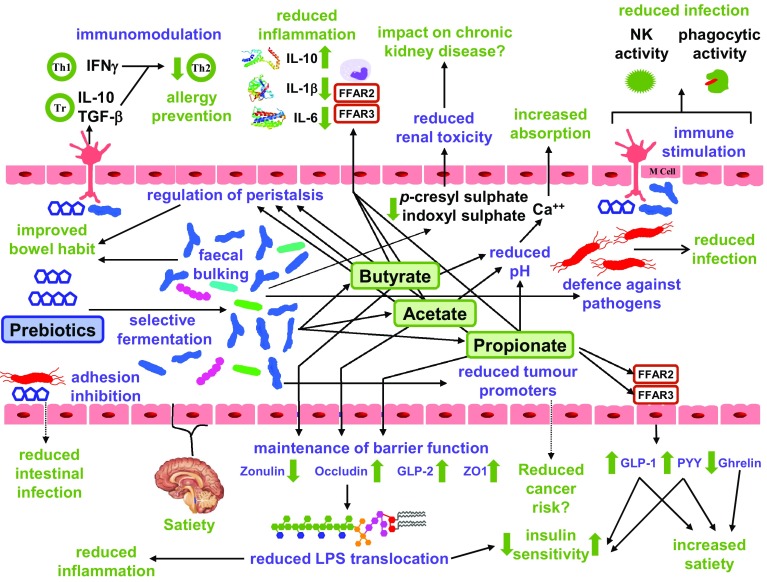
Effect of prebiotics on gut function and health. The figure indicates likely mechanism of prebiotic action in the gut. In many cases the suggested mechanisms are speculative at the present time. Physiological functions are in purple and health outcomes are in green. Abbreviations: FFAR2/GPR43, free fatty acid receptor 2; FFAR3/GPR41 free fatty acid receptor 3; GLP-1, glucagon-like peptide 1; GLP-2, glucagon-like peptide 2; IFN-γ, interferon gamma; IL-1β, interleukin 1 beta; IL-6, interleukin 6; IL-10, interleukin 10; LPS, lipopolysaccharide; NK, natural killer cells; PYY, peptide YY; Th, T helper cells; TGF-β, transforming growth factor beta; Tr, T regulatory cells; ZO-1 zona occuldens protein 1

Figure 2 summarizes the effects of prebiotics on human health. Several studies have examined the effect of prebiotics on allergic reactions and infections in infancy. A placebo-controlled randomized trial of infants with a parental history of atopy showed that formula milk supplemented with a prebiotic mixture of galacto-oligosaccharides (GOS) and long chain inulin significantly reduced the incidence of atopic dermatitis. Prebiotic supplements were associated with a significantly increased number of fecal bifidobacteria, but with no significant change in lactobacilli numbers [17]. In this same cohort of infants, the prebiotic supplemented milk significantly reduced the incidence of infectious episodes during the first 6 months of life [18]. In a 2-year follow-up study of this cohort, infants receiving prebiotic supplementation had a significantly lower incidence of allergic manifestations [19]. At 5-year follow-up, infants in the prebiotic supplementation group had a significantly lower incidence of any allergic manifestation and atopic dermatitis compared to the placebo group [20]. The proposed mechanism for this long-lasting effect of prebiotics is immune modulation mediated through changes in the intestinal microbiota [19]. In a three-group randomized intervention study, infants fed prebiotic GOS+inulin supplemented milk had comparable numbers of fecal bifidobacteria and lactobacilli to infants who were breast fed, whereas infants fed standard formula milk had significantly lower numbers of both bacterial genera. Incidence of gastrointestinal and upper respiratory tract infections was significantly lower in breast fed infants or the ones fed prebiotic supplemented milk compared to standard formula milk. Similarly, allergic reactions to food and milk were significantly higher in the standard formula milk group [21].

A meta-analysis of 26 randomized controlled trials (RCTs) involving 831 healthy adults showed that dietary prebiotic supplementation significantly increased self-reported feelings of satiety compared with placebo [22]. Healthy adults fed an oligofructose-enriched inulin diet experienced lowered hunger and increased satiety rates compared with the placebo, maltodextrin. The increased feeling of satiety was accompanied by an increase in plasma gut peptide concentrations of glucagon-like peptide 1 (GLP-1) and peptide YY in prebiotic supplemented subjects, which may have contributed to the change in appetite [23], suggesting a potential for use in treating obesity. Similarly, in obese or overweight children, an oligofructose-enriched inulin diet significantly increased satiety compared with maltodextrin. Prebiotic supplementation led to a significant reduction in energy intake in older (aged 11–12 years), but not younger (aged 7–10 years) children [24] suggesting that prebiotic supplementation can potentially help to regulate energy intake in obese children.

Prebiotics have been used in several studies to treat constipation. A meta-analysis of RCTs involving 252 subjects (experimental group: *n* = 144, control group: *n* = 108) reported that inulin significantly improved bowel function in patients with chronic constipation exhibiting beneficial effects on stool frequency, the Bristol scale of stool consistency, transit time and stool hardness [25]. Following an evidence review the European Food Safety Authority (EFSA) concluded that “chicory inulin contributes to maintenance of normal defecation by increasing stool frequency” [26]. The results were recently confirmed in a randomized, placebo-controlled study showing that chicory inulin was effective in treating healthy subjects with constipation, increasing stool frequency significantly compared with placebo [27].

Additional described effects of prebiotics include reducing toxins produced from protein metabolism in urine (*p*-cresol and ammonia) [28] and serum (*p*-cresyl sulphate) [29], and increasing calcium absorption in adolescents [30, 31]. Prebiotics may also exert beneficial effects on host physiology which are independent of the microbiota as demonstrated by in vitro experiments for GOS. These included modulation of goblet cells to enhance mucosal barrier function [32], a direct protective effect on intestinal barrier function [33], and inhibiting adherence of enteropathogenic *Escherichia coli* to Caco-2 enterocyte and Hep-2 epithelial cells [34].

An improved understanding of the functional ecology of the gut and a more detailed knowledge of gut metabolites are particularly important for understanding the role of prebiotics on human health. For some products there is already good evidence on gut health and these findings should be communicated to health care professionals and consumers. On the other side, more studies on the effect of prebiotics on health outcomes in humans are imperative.

## The intestinal microbiome: a clinical perspective

The human gut microbiota consists of trillions of microbes which form a complex ecosystem [35]. Although, some researchers have suggested that the number of microbes in the human gut is tenfold the total number of human somatic cells, a recent estimate has calculated that the numbers are of the same order, with the total number of bacteria in the human body being around 3.8 × 10^13^ [36]. An aberrant gut microbiota has been described in several disorders including IBS, with exogenous factors such as antibiotics also causing disturbance of the intestinal microbiota [35].

The systemic effect of microbiota is mediated by microbial metabolites such as short-chain fatty acids (SCFAs), and the gases hydrogen sulfide, ammonia, hydrogen, methane, carbon monoxide and carbon dioxide [37, 38]. SCFAs, which comprise mainly acetate, propionate and butyrate, are produced under anaerobic conditions in the large intestine by fermentation of dietary fibers [37]. SCFAs activate the G protein-coupled receptors, GPR41/FFAR3 (free fatty acid receptor 3) and GPR43/FFAR2, which are present on multiple cell types including intestinal epithelial cells, macrophages, dendritic cells and mast cells [37, 39, 40]. Consequently, SCFAs have multiple effects on the host, including acting as an energy source, promoting glucose and energy homeostasis, regulating immune responses and inflammation, regulating anorectic hormones which have a role in appetite control, tumor suppression (especially butyrate), and regulating central and peripheral nervous systems [37, 39–42].

The effects of butyrate on the human colonic mucosa were examined following administration of butyrate enemas at physiologically relevant concentrations in healthy volunteers. Transcription analysis of microbiome revealed that butyrate induced differential expression of multiple genes involved in fatty acid oxidation, electron transport chain and oxidative stress pathways [43]. In addition, butyrate led to dose-dependent decreases in visceral sensitivity [44]. However, butyrate enemas administered to patients with UC in clinical remission had relatively minor effects on inflammatory and oxidative stress parameters, although the selection of patients with chronically mild levels of inflammation and oxidative stress may have limited the scope of this study [45].

Protection against microbial invasion is provided by the intestinal barrier [46]. The intestinal barrier has multiple lines of defense including commensal bacteria, which competitively inhibit the colonization of pathogenic bacteria and the production of metabolically protective compounds such as butyrate [46]. Impaired intestinal barrier function may result in a local or systemic immune response, mast cell degranulation, neuroinflammation and afferent vagus nerve activation [46]. In addition, commensal bacterial species such as *Lactobacillus plantarum* regulate intestinal epithelial integrity by stimulation of Toll-like receptor 2 (TLR2) in the gut epithelium [47]. In one study, extensive transcriptome analysis following consumption of three probiotic strains, *Lactobacillus acidophilus, L. casei*, and *L. rhamnosus*, by healthy volunteers showed that each species induced differential gene expression in networks involved in regulation of major basal pathways in the small intestinal mucosa, which resembled those induced by specific bioactive molecules and drugs [48]. The potential of probiotic bacteria to improve intestinal barrier function is discussed extensively in a recent review [49].

Investigation of intestinal barrier function and intestinal permeability can be done by using a so-called Ussing chamber, an ex vivo method that uses intestinal specimens. The multi-sugar test is a non-invasive method that measures urinary excretion of ingested sugars as a measure of gut permeability [50, 51]. Indicators for gastroduodenal and small intestinal permeability are sucrose excretion and the lactulose/rhamnose ratio in 0–5 h, respectively. Colonic permeability is estimated by the sucralose/erythritol ratio from urine sampled 5–24 h after the sugar ingestion. Application of the multi-sugar test showed that small intestinal permeability was increased in patients with diarrheal IBS compared to healthy controls [50].

Patients with post-infectious IBS have reduced mucosal and fecal microbial diversity compared with healthy controls. In addition, the intestinal microbiota of post-infectious IBS patients was shown to be different from that of general IBS patients [52]. Differences between post-infectious IBS patients and healthy controls were also found with respect to release of immunoregulatory cytokines (IL-13, IL-10 and IL-1β) following ex vivo stimulation of colonic biopsies with selected species of anaerobic commensal bacteria. These results are consistent with an altered immune response against commensal gut microbes in post-infectious IBS patients [53].

Therapeutic alteration of intestinal microbiota in conditions such as IBS may be achieved by ingestion of probiotics and prebiotics to increase the number of commensal bacteria within the gut, antibiotics which deplete pathogenic bacteria, and fecal microbiota transplantation (FMT) which introduces a healthy, diverse microbiota into the gut [35]. A meta-analysis of FMT reported that the method was highly effective for treating recurrent *Clostridium difficile* infection [54] and an expert consensus panel has recommended indications, technical procedures and clinical trial details of FMT for treating various conditions [55]. The panel also considered that, at the present time, FMT should be performed only in research settings for treatment of IBD, IBS and metabolic syndrome [55]. Further research is needed to establish the role of FMT for treating these disorders.

## Microbiome-nutrient interactions in the diseased gut

LifeLines is a large prospective cohort study in The Netherlands that includes more than 165,000 individuals, representing three generations, with a proposed duration of 30 years. The study collects extensive data on participants including demographic, biological and phenotypic information including genetic, epigenetic and ‘omics’ data (metabolomics, transcriptomics, proteomics), with a wide range of biomaterials stored in a biobank. Subjects are required to complete a questionnaire each year, and several biomarkers are measured every 5 years [56]. LifeLines Deep is a cohort of 1500 individuals within LifeLines for whom multiple layers of omics information have been generated including both 16S and whole genome metagenomic sequence data [57]. At the time of the symposium, full metagenomic sequence data was available from ~ 1600 population-based individuals including approximately 1100 of the LifeLines DEEP population [57] and ~ 500 from the Functional Genomics Project [58, 59]. In addition, two disease focused cohorts are also available including 380 patients with IBD and 400 patients suffering from IBS [57, 60–63].

Genetic analysis of the human gut microbiota is commonly performed by high-throughput metagenomic sequencing and taxonomic profiling following analysis of 16S ribosomal RNA gene sequences [64]. Full metagenomic sequencing of isolates enables not only taxonomic profiling, but also can gain insight at the strain level, and into functional parameters such as metabolic pathways and other biological processes, virulence factors, and antibiotic resistance. However, there is still a limited understanding of individual factors that shape the microbiota on individual level.

It is known that the overall diversity of the human gut microbiota changes throughout life, increasing steadily from birth until around 12 years of age, remaining relatively stable throughout adulthood, and then declining in later years [65]. In adults, 60–70% of the gut microbiome is stable, with the degree of stability varying between phyla [66]. Infections, lifestyle and dietary changes cause microbiome instability, producing major perturbations of the gut microbiome as nicely shown in a high-resolution longitudinal study in two individuals [67]. To study the role of the gut microbiome in health and disease, the scientific world first must address the question: what is a “healthy” microbiome and which factors influence the gut microbiome composition. For addressing this question and defining the intrinsic and extrinsic factors that influence the gut microbiome, Zhernakova et al analyzed the LifeLines Deep cohort utilizing metagenomic shotgun sequencing of the gut microbiome of 1135 participants and more than 200 phenotypic features. This study highlighted a relationship between the microbiome and multiple extrinsic and host factors, comprising 60 dietary factors, 31 intrinsic factors, 19 drug categories, 12 diseases, and 4 smoking categories. Together, these factors accounted for 18.7% of the observed inter-individual variation in the gut microbiome with diet being a major modulator of gut microbiome variation [63].

Multiple intrinsic factors that were associated with inter-individual variation in the gut microbiome included chromogranin A, a member of the granin family of neuroendocrine secretory proteins, stool frequency and Bristol classification of stool type but interestingly also triglyceride concentrations. Age and high-density lipoprotein (HDL) concentration were positively correlated with gut microbiome inter-individual variation [63]. In another study, our group showed by performing a Mendelian Randomization study that the human gut microbiota is an independent factor for variation of blood lipid levels, accounting for 6% of triglyceride, and 4% of HDL variance. In addition, we could show that 4.5% of the variance in BMI is attributable to the gut microbiome [68].

Analysis of the gut microbiome revealed that the use of proton pump inhibitors (PPIs) was associated with a significant decrease in gut microbiota diversity and with significant changes of around 20% of bacterial taxa. This adverse effect of PPIs on bacterial diversity was greater than for any other drug class, including antibiotics. PPIs depleted beneficial bacteria such as the Ruminococcaceae family and *Bifidobacterium*, and increased potentially harmful bacteria including *Enterococcus, Streptococcus, Staphylococcus* genera and *Escherichia coli*. Results suggested that PPIs diminished the gastric acid barrier, as species found in the oral microbiome of PPI users were more abundant in the gut than in non-users [61]. It is increasingly observed that the use of PPIs is associated with an increase in the incidence of enteric infections like *Clostridium difficile* and *Campylobacter*. Given the profound effect of PPIs on the gut microbiome and the fact that over 11% of population in The Netherlands and other European countries are using PPIs on prescription (not including over the counter use of PPIs) implies a major PPI-dependent influence on the gut microbiome taxonomy and function on a populational scale.

The Microbiome working group within the UMCG has embarked recently on a large project within the LifeLines cohort: the 10K metagenome project. Full metagenomic sequence data will be generated from fresh frozen fecal samples of 10,000 individuals. In addition to the genetic data, more than 2000 phenotypic details will be available for each individual. It is planned that the subjects will prospectively followed up every 5 years.

Taken together, the population-based LifeLines cohort is providing valuable insight into the complex interaction of microbiome with human health and will be instrumental in outlining new biomarkers and treatments for human diseases.

## Effects of vitamins on the microbiome

The human gut microbiota contains bacteria that are beneficial to the host, and bacteria with pathogenic potential, termed ‘pathobionts’ [69]. An important role of beneficial bacteria is the metabolic production of SCFAs by cross-feeding (Fig. 1). Fiber-degrading bacteria include *Ruminococcus callidus, Ruminococcus albus, Blautia obeum and Prevotella* spp. which produce solubilized oligosaccharides and polysaccharides that act as substrates for butyrate-producing species such as *Faecalibacterium prausnitzii, Eubacterium rectale, Roseburia* spp, *Eubacterium hallii* and *Anaerostipes spp* [37]. Butyrate has multiple effects on the host including maintenance of gut barrier function by stimulating the production of mucin, antimicrobial peptides, and tight-junction proteins and reducing colonic oxidative stress [70]. These effects on gut barrier function are important for health as changes in the mucosal barrier have been described in IBD [71].

Gut microbiota imbalance, or dysbiosis, is considered to play a significant role in the pathogenesis of intestinal disorders such as IBD and IBS, and of extra-intestinal disorders including allergies, asthma, type 1 diabetes, cardiovascular disease, metabolic syndrome, and obesity [72]. Chemotherapy-induced mucositis which occurs in the mouth and gut results from damage to the mucosal barrier and can result in bacteremia, which is the abnormal presence of bacterial in blood. It has been suggested that commensal intestinal bacteria may play a key role in amelioration of inflammation and bacteremia [51]. In a rat model of chemotherapy-induced mucositis, the number and diversity of the fecal microbiota was substantially decreased, including anaerobes and Streptococci, although there was a relative increase of Bacteroides [73]. Supporting the beneficial anaerobic microbiota during chemotherapy may, therefore, improve treatment and quality of life for cancer patients.

**Fig. 3:**
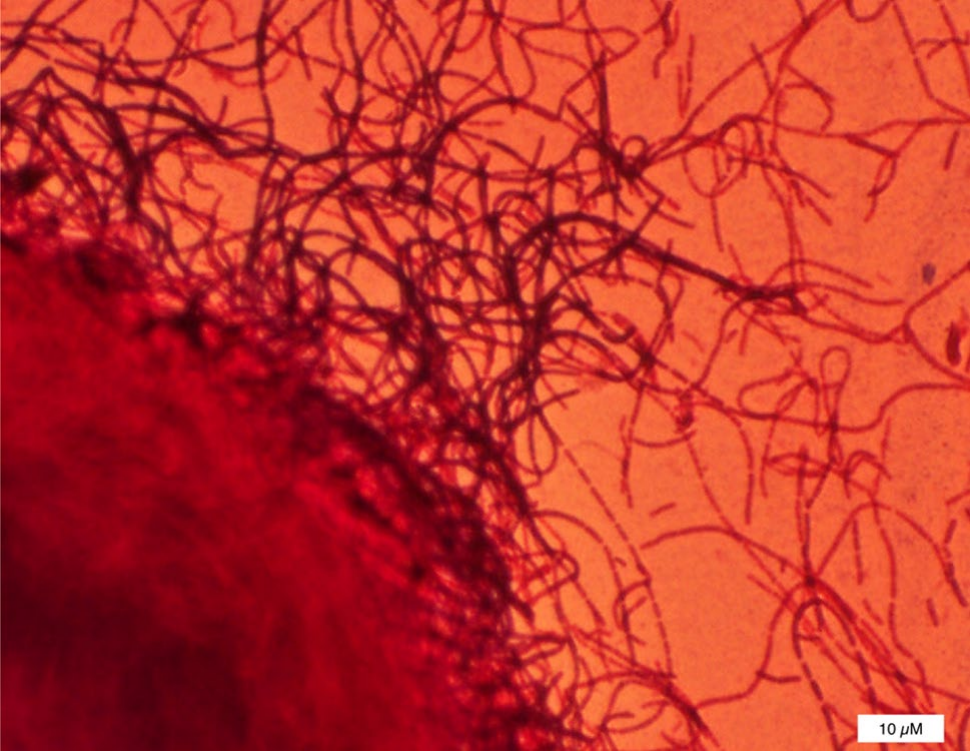
A picture of the Gram-stained cells of *Faecalibacterium prausnitzii* growing in a colony inside agar seen as a big ball at the left lower corner, chains of cells grow away from this colony [80]. A typical single cell has the size of 3–5 µm in length and 1 µm in diameter. The bar represents 10 µm (Photo, M. Sadaghian Sadabad)

*Faecalibacterium prausnitzii* is a Gram-negative obligate anaerobe which is difficult to culture, and taxonomically is in the Clostridia order of Firmicutes (Fig. 3). It is present in the gut of all healthy humans and may act as a biomarker of a healthy gut [74]. Dysbiosis associated with CD is characterized by reduced abundance of *F. prausnitzii* [75], with dysbiotic ileal CD patients having a significantly lower abundance of *F. prausnitzii* and a concomitantly increased abundance of *E. coli* [76]. Mechanistically, animal experiments provide an explanation for the increased abundance of *E. coli* in IBD as nitrate, which was generated as a byproduct of the inflammatory host response, selectively enhanced growth of *E. coli* in the large intestine of mice [77]. Moreover, *F. prausnitzii* produces a 15 kDa anti-inflammatory protein that inhibits the NF-κB pathway in intestinal epithelial cells and was shown to prevent colitis in a mouse model [78].

*F. prausnitzii* uses riboflavin (vitamin B2) as a mediator for extracellular electron transfer, as demonstrated in a microbial fuel cell system [79]. A Human oxygen-Bacteria anaerobic (HoxBan) co-culture system has been developed in which *F. prausnitzii* was cultured with adherent Caco-2 cells. Caco-2 cells promoted the growth and metabolism of the anaerobic *F. prausnitzii*, while genes involved in inflammation and oxidative stress in Caco-2 cells were suppressed by *F. prausnitzii* [80].

Anti-oxidants including riboflavin and vitamin C are being investigated as new targets for intervention for the treatment of dysbiosis. A first pilot open-label study with 100 mg/day riboflavin showed indeed an increase in faecalibacteria and a reduction in *E. coli* in most participants [81]. The double-blind, parallel-group, placebo-controlled Ribogut trial is currently examining the effect of 50 or 100 mg/day riboflavin administered to healthy volunteers for 14 days on the gut microbiota composition with results to be expected in 2018.

## Early-life development of a healthy microbiome

**Fig. 4:**
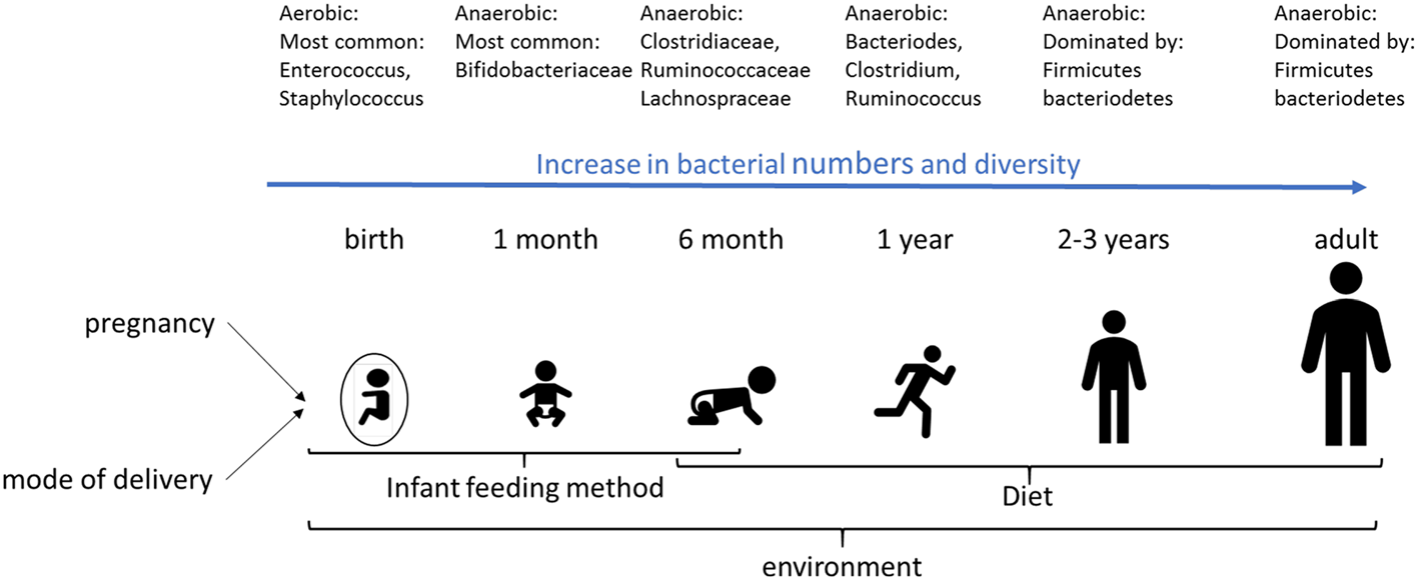
Development of the gut microbiome during infancy. The development of the infant microbiome is dependent on various factors, such as infant feeding method, diet and the environment. Also, the mode of delivery (either vaginal or by cesarean section) affects the early life microbiome. Transfer of bacteria from the mother to the fetus has also been shown, indicating that pregnancy may be important for colonization of the fetal/infant gut

The development of the perinatal gut microbiota is influenced by multiple factors including gestational age, mode of delivery, maternal microbiota, infant feeding method, genetics, and environmental factors such as the choice of food. Microbial diversity increases dramatically during first months of infancy (Fig. 4). At birth, the microbiota is aerobic, with low numbers and low diversity, with the most common bacteria facultative anaerobes and members of the Enterobacteriaceae phylum [82]. Within a few days, the gut environment becomes anaerobic resulting in growth of bacteria such as Bifidobacterium [82], which is the dominant bacterium genus in the infant gut in the first months of life. With the introduction of solid food, a more adult-like microbiome starts to develop as of 6 months of life, dominated by Firmicutes and Bacteriodetes [82].

Factors promoting a healthy microbiota in neonates include a vaginal delivery, delivery at term, breast feeding, and exposure to a variety of microorganisms. In contrast, a Caesarean section, premature delivery, formula milk, and exposure to antibiotics have a negative impact on the diversity and composition of microbiota in infants [25, 83–85].

Preterm infants show delayed colonization of the gut microbiota with *Bifidobacterium*, and have a high prevalence of Enterobacteriaceae, *Staphylococcus*, and Enterococcaceae [25]. Vaginally delivered neonates have an increased prevalence of maternal microbiota derived from the vagina and intestine (e.g. *Lactobacillus, Prevotella* and *Sneathia*) compared with neonates delivered by Caesarean section. Caesarean section delivered infants have a relatively high prevalence of skin bacteria such as *Staphylococcus, Propionibacterium* and *Corynebacterium* compared to the ones that are vaginally delivered [25, 84]. Maternal antibiotic treatment that results in reduced utilization of human milk and prolonged hospitalization normally causes an increased prevalence of Proteobacteria, Firmicutes, Enterobacteriaceae (*E. coli* and *Klebsiella* spp.), *Staphylococcus, Propionibacterium* and *Corynebacterium* [25]. Feeding formula milk is associated with increased bacterial diversity, increased prevalence of *Bacteroides fragilis, Clostridium difficile*, and *E. coli*, and a decreased prevalence of bifidobacteria [84].

As previously mentioned, dysbiosis in infancy is associated with an increased risk for immunological diseases such as asthma, allergic rhinitis, type 1 diabetes and celiac disease in addition to metabolic diseases, e.g. obesity and type 2 diabetes [84, 85].

Pregnancy is shown to alter the maternal gut microbiota. In humans, dramatic changes in the gut microbiota during pregnancy were described from the first to third trimesters with an overall increase in the relative abundance of Proteobacteria and Actinobacteria, and with a reduced richness with a decreased abundance of Firmicutes and Bacteroidetes [86]. In C57BL/6 mice, pregnancy produced significant increases in the relative abundance of genera including *Akkermansia, Bacteroides, Bifidobacterium*, and *Clostridium*, in comparison to non-pregnant females. Changes in the microbiota began at the onset of pregnancy [87]. Pregnant Balb/c mice showed significant increases in the relative abundance of Actinobacteria and Proteobacteria compared with non-pregnant littermates. The relative changes in gut microbiota of non-pregnant and pregnant mice were strain-specific suggesting that genetic background is an important determinant of the microbiome. The physiological changes that occur in pregnancy produces significant changes in maternal metabolism necessary for supporting a healthy pregnancy [86]. The mechanisms resulting in alteration of the microbiota during pregnancy are largely unknown, but it seems likely that microbiome alterations during pregnancy are also important to support changes in maternal immune status and/or hormonal changes [86, 88].

It has also been suggested that changes in the maternal microbiota during pregnancy are important for fetal health, since it has been shown that maternal microbiota can be transferred to the fetus [89]. The transfer of microbiota from the mother to the fetus can be observed in the meconium, which in contrast to the earlier beliefs is not sterile [89]. The microbiota of meconium has low diversity represented by the Firmicutes (*Staphylococcus, Enterococcus*, and *Bacilli*), Proteobacteria and Actinobacteria phyla, and low bacterial cell numbers [90]. Variation in the microbiota of meconium is affected by maternal diabetes status [91] and maternal gestational diet, with a high-fat diet producing pronounced changes in neonatal meconium which persisted in infant faces for up to 6 weeks of age [92].

Also, the human placenta is not sterile. Indeed, the placental microbiome is unique and is comprised of commensal bacteria from the Firmicutes, Proteobacteria, Bacteroidetes, Tenericutes, and Fusobacteria phyla, and has some similarity to the human oral microbiome [93]. Low diversity of placental microbiota was significantly associated with low birth weight in full-term neonates [94]. Inter-individual placental microbiome diversity (beta diversity) was significantly associated with prenatal infection or a preterm birth [93].

Direct evidence of transfer of maternal bacteria is derived from experiments in mice, in which oral administration of a genetically-labelled *Enterococcus fecium* strain to pregnant mice, resulted in a subsequent detection in meconium [89]. Furthermore, experiments of microbial colonization of pregnant germfree mice demonstrated that maternal microbiota affects neonatal immune responses. Gestational colonization had effects on the innate intestinal immune response of the offspring, with increased numbers of intestinal innate lymphoid cells (ILC3), macrophages and dendritic cells, in addition to an effect on intestinal gene expression including genes involved in pathways for sugar metabolism, epithelial cell division and proliferation, and mononuclear cell function [95].

The development of the neonatal microbiome is dependent on various factors. It is known since long that birth mode, feeding mode and antibiotic exposure, all affect the development of the neonatal microbiome. Since also treatment with pre- or probiotics may affect the neonatal microbiome, such treatments may be effective options to optimize development of the neonatal microbiome.

It has become clear that the fetus and placenta are not sterile and the transfer of bacteria occurs from mother to the fetus during pregnancy. Therefore, the maternal microbiome also seems to be important for the development of the neonatal microbiome. This implies that pre- or probiotics use may open a possibility to modulate the maternal microbiome during pregnancy, to optimize the development of the fetal microbiome. Further studies on the role of the maternal microbiome in development of the neonatal microbiome are necessary.

## Microbiome and the gut-brain axis

The bidirectional signaling between the gut microbiota, the gut, and the brain occurs via neuronal pathways involving both the central and enteric nervous systems in addition to the circulatory system [96, 97]. The latter includes involvement of the hypothalamic–pituitary–adrenal (HPA) axis, immune system regulators, hormones, bacterial metabolites such as SCFAs, and neurotransmitters [96, 98].

Preclinical studies have shown effects of the gut microbiota on nociceptive reflexes [99], feeding, emotional and social behavior [99], the stress response [99], and brain neurochemistry [100, 101]. The gut microbiota is essential for normal social development in the mouse and is implicated in neurodevelopmental disorders including autism spectrum disorder [102–104]. Germfree mice have an exaggerated stress response compared with control animals. These mice also exhibit increased motor activity and lower anxiety-like behavior compared with control mice [105]. Administration of the probiotic *L. rhamnosus (JB-1)* to mice reduced stress-induced corticosterone levels and anxiety-related behavior [101]. These data strongly highlight the importance of the microbiome-gut-brain axis for normal neurological development and function.

Central control of the gut is mediated through the HPA axis and the autonomic nervous system. Preclinical studies on the stress response illustrate the effect of the CNS on the gut microbiome [97]. In primates, prenatal and postnatal stress affected the composition of the intestinal microbiota [106]. In addition to changes in the microbiome, postnatal stress was associated with stress-indicative behavior [106]. In rats, postnatal stress altered the fecal microbiome, with notable changes in behavior and immune status [107].

The mechanisms by which the gut microbiota exert their effects on the brain are beginning to be understood [97]. Circulating SCFAs produced by gut microbiota influence the integrity of the blood–brain barrier (BBB) by increasing production of the tight junction proteins claudin-5 and occludin. This increased BBB integrity limits entry of undesirable metabolites into brain tissue [98]. Compounds collectively known as microbe-associated molecular patterns (e.g. lipopolysaccharide, bacterial lipoprotein, flagellin and CpG islands of unmethylated DNA) produced by the gut microbiota influence neuroimmune function by stimulating the release of cytokines such as TNFα, IL-6 and IL-1β from innate immune cells such as dendritic cells, macrophages and neutrophils. These cytokines can cross the BBB and activate microglia and neurons resulting in altered neurological function which can result in a change in mood and behavior [98].

A growing number of placebo-controlled RCTs have investigated the effect of probiotics on mood, cognition and brain function in humans. In healthy women, ingestion of a fermented milk product supplemented with probiotics containing *Bifidobacterium animalis*, subsp. lactis, *Streptococcus thermophiles*, and two *Lactobacillus* spp. produced significant changes in brain activity assessed by functional magnetic resonance imaging (fMRI), in response to an emotional faces attention task. Reduced fMRI reactivity was found in interceptive and somatosensory regions of the brain which control central processing of emotion and sensation [108]. Probiotics containing *Lactobacillus helveticus* and *Bifidobacterium longum* showed beneficial psychological effects in healthy human volunteers, with significant improvements in several global tests including the reduction of global psychological symptoms, depression and anxiety [109]. A probiotic milk drink containing *Lactobacillus casei* Shirota ingested by healthy volunteers had no effect on the mood of the group overall, but improved mood in subjects with low baseline mood, although an unexpected finding was somewhat impaired performance on two memory recall tests [110]. Consumption of a multispecies probiotic containing two *Bifidobacterium* spp and five *Lactobacillus* spp. by healthy participants produced a significant reduction in overall cognitive reactivity (negative thoughts) to sad mood [111].

A link between the gut and brain function is supported by additional human studies involving diseased or normal subjects. Many alcohol-dependent subjects have alterations in their intestinal permeability and gut microbiome. Increased intestinal permeability in these subjects was significantly associated with higher scores of depression, anxiety, and alcohol craving following 3 weeks of abstinence [112]. A placebo-controlled RCT of patients with major depressive disorder showed that a probiotic containing two *Lactobacillus* spp. plus *Bifidobacterium bifidum* produced a significant decrease in Beck Depression Inventory total scores, significant decreases in serum insulin levels and serum high-sensitivity C-reactive protein (hs-CRP) concentrations in addition to a significant increase in plasma total glutathione concentrations [113]. A RCT of patients with Alzheimer’s disease found that ingestion of a probiotic containing three *Lactobacillus* spp. plus *Bifidobacterium bifidum* significantly improved the mini-mental state examination scores, and produced significant changes in a range of metabolic parameters including plasma malondialdehyde, serum hs-CRP and serum triglycerides [114]. Consumption of the prebiotic B-GOS, but not the prebiotic FOS, by healthy volunteers significantly reduced the salivary cortisol awakening response when compared to placebo [115].

Thus, these and similar studies provide the evidence that the gut microbiota can modulate the stress response and is also implicated in anxiety, depression and cognition. Therefore, the introduction of probiotic or symbiotic nutritional approaches are put forward by researches to prevent, delay, or ease neurological disorders in the future (see: [97]). However, the underlying mechanisms of these interactions are largely unclear and, at the present time, it is not possible to differentiate between the microbes involved.

## Conclusions

The role of the human gut microbiota in health and disease is beginning to be understood. The composition of the gut microbiota is influenced by intrinsic mechanisms such as stress, and extraneous factors such as diet, prebiotics, probiotics, and drugs including PPIs and antibiotics. The dysbiosis of gut microbiota has been shown to be associated with IBD, IBS and depression.

It is clear that the gut microbiota is active, not passive, in its relationship with its host. Microbial metabolites (such as SCFAs) affect gut-brain signaling. The gut microbiota has a regulatory role on anxiety, mood, cognition and pain which is exerted via the gut-brain axis. In pregnancy dramatical changes of the maternal microbiota affects neonatal immune responses and maturation.

Ingestion of prebiotics or probiotics has been used to treat a range of conditions including constipation, allergic reactions and infections in infancy, and in patients with IBS. FMT is highly effective for treating recurrent *Clostridium difficile* infections and may be used more widely in the future for conditions such as metabolic syndrome.

Taken together, the effects of gut microbiome on health are multifaceted and researchers and health professionals try to educate consumers by including new scientific information into their practice, especially for benefits beyond digestive health. It is, therefore, expected that pre/probiotics will be combined with other nutritional compounds to achieve a more robust health effect. Moreover, it is expected that combining different research disciplines and utilization of new technological methodologies in the microbiome research may pave the way for developing evidence-based clinical interventions for health concerns of modern life.
